# Outcomes of Pediatric SARS-CoV-2 Omicron Infection vs Influenza and Respiratory Syncytial Virus Infections

**DOI:** 10.1001/jamapediatrics.2023.5734

**Published:** 2023-12-26

**Authors:** Pontus Hedberg, Lina Abdel-Halim, John Karlsson Valik, Tobias Alfvén, Pontus Nauclér

**Affiliations:** 1 Department of Medicine Huddinge, Karolinska Institutet, Stockholm, Sweden; 2 Department of Infectious Diseases, Karolinska University Hospital, Stockholm, Sweden; 3 Department of Global Public Health, Karolinska Institutet, Stockholm, Sweden; 4 Division of Infectious Diseases, Department of Medicine, Solna, Karolinska Institutet, Stockholm, Sweden

## Abstract

This cohort study compares outcomes of SARS-CoV-2 Omicron infection with those of influenza or respiratory syncytial virus infection in pediatric patients attending the emergency department.

Pneumonia is the largest infectious cause of pediatric mortality, and respiratory viruses, including respiratory syncytial virus (RSV), influenza virus, and SARS-CoV-2, can be detected in more than 80% of community-acquired infections.^1,2^ Omicron is associated with less severity compared with preceding variants.^3^ However, outcomes of Omicron vs RSV and influenza infections in children remain to be better understood. We compared outcomes of Omicron infection with those of influenza or RSV infection in pediatric patients attending emergency departments (EDs).

## Methods

This multicenter, retrospective cohort study used 5 population-based data sources and included all 3 pediatric EDs in Stockholm, Sweden, covering approximately 500 000 individuals younger than 18 years. Informed consent was waived by the Swedish Ethical Review Authority, which approved the study, because analyses were based on retrospectively collected data. The study followed the STROBE reporting guideline. We identified individuals younger than 18 years attending the ED from August 1, 2021, to September 15, 2022, with a polymerase chain reaction (PCR) test positive for SARS-CoV-2, influenza A/B, or RSV from 1 day before to 1 day after the ED visit. Multiplex PCR testing of all 3 viruses was introduced February 2021, and more than 99% of the study population was tested for all 3 viruses. For the cohort with Omicron, only visits from December 27, 2021, onward were included, a period when Omicron was the dominating variant (>99% of sequences from Stockholm). We excluded patients testing positive for more than 1 virus. Two infectious diseases physicians (J.K.V., P.N.) reviewed diagnoses to include visits likely due to respiratory infections (eTable 1 in Supplement 1). The first visit meeting these criteria was included. Outcomes were hospitalization, intensive care unit (ICU) admission, and 30-day all-cause mortality. Hospitalizations with admission due to infection were considered (eTable 2 in Supplement 1). Logistic regression models adjusted for age, sex, and comorbidities were used to compare hospitalizations for RSV and influenza vs Omicron. Variables are described in eTable 3 in Supplement 1. R, version 4.1.0, was used for analysis.

## Results

We included 2596 pediatric patients (896 [34.5%] with Omicron, 426 [16.4%] with influenza A/B, and 1274 [48.0%] with RSV). Of patients with RSV, 990 (77.7%) were younger than 2 years vs 648 (72.3%) with Omicron and 81 (19.0%) with influenza (Table 1). Hospitalization rates were 31.5% (n = 282) for Omicron, 27.7% (n = 118) for influenza, and 81.7% (n = 1041) for RSV (Table 2). For infants aged 0 to 1 year, odds ratios (ORs) for hospitalization were 11.29 (95% CI, 8.91-14.38) for RSV vs Omicron and 1.67 (95% CI, 1.03-2.68) for influenza vs Omicron. For children aged 2 to 4 years, ORs were 3.96 (95% CI, 2.25-7.01) and 0.31 (95% CI, 0.15-0.65), respectively. For youths aged 5 to 17 years, ORs were 5.22 (95% CI, 2.40-11.81) and 1.10 (95% CI, 0.69-1.77), respectively. ICU admission rates were 0.7% (n = 6) for Omicron, 0.9% (n = 4) for influenza, and 2.9% (n = 37) for RSV. Three patients died within 30 days: 2 (0.2%) with Omicron and 1 (0.1%) with RSV.

**Table 1.** Characteristics of and Outcomes in Cohorts With SARS-CoV-2 Omicron, Influenza, or RSV Infection^a^

Characteristics	Age 0-1 y			Age 2-4 y			Age 5-17 y		
	Omicron (n = 648)	Influenza (n = 81)	RSV (n = 990)	Omicron (n = 81)	Influenza (n = 80)	RSV (n = 236)	Omicron (n = 167)	Influenza (n = 265)	RSV (n = 48)
Sex									
Female	306 (47.2)	45 (55.6)	440 (44.4)	34 (42.0)	46 (57.5)	113 (47.9)	77 (46.1)	124 (46.8)	21 (43.8)
Male	342 (52.8)	36 (44.4)	550 (55.6)	47 (58.0)	34 (42.5)	123 (52.1)	90 (53.9)	141 (53.2)	27 (56.2)
Age, median (IQR), y	0.0 (0.0-0.0)	0.0 (0.0-0.0)	0.0 (0.0-0.0)	3.0 (2.0-3.0)	3.0 (2.0-4.0)	2.0 (2.0-3.0)	10.0 (7.0-13.5)	10.0 (7.0-14.0)	7.0 (5.0-10.0)
Comorbidities									
Asthma	6 (0.9)	4 (4.9)	20 (2.0)	19 (23.5)	23 (28.7)	74 (31.4)	39 (23.4)	60 (22.6)	20 (41.7)
Cancer	1 (0.2)	0	0	6 (7.4)	2 (2.5)	2 (0.8)	12 (7.2)	5 (1.9)	6 (12.5)
Cardiac disease	5 (0.8)	0	6 (0.6)	1 (1.2)	1 (1.2)	7 (3.0)	4 (2.4)	6 (2.3)	3 (6.2)
Chronic kidney disease	0	0	0	1 (1.2)	0	0	0	2 (0.8)	1 (2.1)
Chronic respiratory disease, not asthma	4 (0.6)	0	4 (0.4)	4 (4.9)	5 (6.2)	6 (2.5)	10 (6.0)	10 (3.8)	18 (37.5)
Congenital malformations and abnormalities	100 (15.4)	10 (12.3)	130 (13.1)	22 (27.2)	22 (27.5)	69 (29.2)	44 (26.3)	64 (24.2)	19 (39.6)
Type 1 or 2 diabetes	0	0	0	1 (1.2)	2 (2.5)	0	4 (2.4)	3 (1.1)	0
Immunocompromised	7 (1.1)	0	1 (0.1)	9 (11.1)	5 (6.2)	13 (5.5)	26 (15.6)	18 (6.8)	10 (20.8)
Perinatal conditions	149 (23.0)	12 (14.8)	218 (22.0)	25 (30.9)	18 (22.5)	75 (31.8)	24 (14.4)	32 (12.1)	11 (22.9)
Any	215 (33.2)	22 (27.2)	304 (30.7)	51 (63.0)	45 (56.2)	158 (66.9)	95 (56.9)	133 (50.2)	39 (81.2)
Outcomes									
Hospital admission	203 (31.3)	34 (42.0)	825 (83.3)	34 (42.0)	17 (21.2)	181 (76.7)	45 (26.9)	67 (25.3)	35 (72.9)
Hospital length of stay, median (IQR), d	2.0 (1.0-3.0)	1.0 (1.0-2.0)	4.0 (3.0-6.0)	2.0 (1.0-2.8)	2.0 (1.0-4.0)	2.0 (1.0-3.0)	3.0 (1.0-5.0)	2.0 (1.0-4.0)	2.0 (1.0-4.0)
High-flow nasal cannula	13 (2.0)	1 (1.2)	350 (35.4)	6 (7.4)	2 (2.5)	15 (6.4)	6 (3.6)	7 (2.6)	6 (12.5)
BiPAP, CPAP, or NIV	0	0	57 (5.8)	0	0	0	2 (1.2)	1 (0.4)	3 (6.2)
ICU admission	2 (0.3)	0	34 (3.4)	1 (1.2)	0	2 (0.8)	3 (1.8)	4 (1.5)	1 (2.1)
Mechanical ventilation	1 (0.2)	0	1 (0.1)	0	0	0	0	0	0
30-d All-cause mortality	0	0	0	0	0	0	2 (1.2)	0	1 (2.1)

Abbreviations: BiPAP, bilevel positive airway pressure; CPAP, continuous positive airway pressure; ICU, intensive care unit; NIV, noninvasive ventilation; RSV, respiratory syncytial virus.

^a^ Data are presented as number (percentage) of individuals unless otherwise indicated.

**Table 2.** Age-Stratified Hospital Admission Rates in the Cohorts With SARS-CoV-2 Omicron, Influenza A/B, or RSV Infection^a^

Age, y	Hospital admissions, No./total No. (%)		
	Omicron (n = 648)	Influenza (n = 81)	RSV (n = 990)
13-17	14/47 (29.8)	22/89 (24.7)	8/11 (72.7)
7-12	26/95 (27.4)	27/115 (23.5)	11/16 (68.8)
5-6	5/25 (20.0)	18/61 (29.5)	16/21 (76.2)
2-4	34/81 (42.0)	17/80 (21.2)	181/236 (76.7)
1	31/79 (39.2)	6/17 (35.3)	118/156 (75.6)
0	172/569 (30.2)	28/64 (43.8)	707/834 (84.8)
Overall	282/896 (31.5)	118/426 (27.7)	1041/1274 (81.7)

Abbreviation: RSV, respiratory syncytial virus.

^a^ Hospitalizations with main diagnosis codes indicative of admission due to respiratory virus infection were considered.

## Discussion

Hospitalization rates were higher in patients infected with RSV vs Omicron in all age groups, but no differences were observed between influenza and Omicron. Patients infected with influenza were older, highlighting difficulties in comparing these patient populations. Hospitalization rates were similar across all age strata likely due to high burden of comorbidities among adolescents and the 2021 to 2022 resurge of influenza and RSV. ICU admissions and mortality were low, as previously observed.^4,5,6^ Strengths include encompassing all pediatric EDs in Stockholm and multiplex PCR testing, favoring internal validity and reducing diagnostic bias. Limitations were the retrospective design with potential underreporting of respiratory support provided and that patients with mild disease might have been missed since they were not tested by PCR. Our findings suggest that RSV infections more often require hospitalization and respiratory support, underscoring the importance of preventive measures, such as recently approved RSV vaccines.

## Notes

Supplement 1. **eTable 1.** Emergency Department *ICD-10* Codes Used for Study Inclusion **eTable 2.** Inpatient *ICD-10* Codes Used for Classification of Hospital Admission **eTable 3.** Study Definitions

Supplement 2. Data Sharing Statement
