# Supplementary material for: Outcomes of Pediatric SARS-CoV-2 Omicron Infection vs Influenza and Respiratory Syncytial Virus Infections
Source: JAMA Pediatr. 2023 Dec 26;178(2):197–9. doi: 10.1001/jamapediatrics.2023.5734 (PMC10751651; doi:10.1001/jamapediatrics.2023.5734)
Supplement: Supplement 1. — eTable 1. Emergency Department ICD-10 Codes Used for Study Inclusion eTable 2. Inpatient ICD-10 Codes Used for Classification of Hospital Admission eTable 3. Study Definitions [file jamapediatr-e235734-s001.pdf]

Supplementary Online Content

Hedberg P, Halim LA, Valik JK, Alfvén T, Nauclér P. Pediatric SARS-CoV-2 Omicron infections compared with influenza and respiratory syncytial virus. *JAMA Pediatr*. 2023;177(12.4):e235734. doi:10.1001/jamapediatrics.2023.5734

eTable 1. Emergency Department *ICD-10* Codes Used for Study Inclusion
eTable 2. Inpatient *ICD-10* Codes Used for Classification of Hospital Admission
eTable 3. Study Definitions

This supplementary material has been provided by the authors to give readers additional information about their work.

© 2023 Hedberg P et al. *JAMA Pediatrics*.

eTable 1. Emergency department ICD-10 codes used for study inclusion

ICD-10 diagnosis code	Diagnosis name
A09.0	Other and unspecified gastroenteritis and colitis of infectious origin
A09.9	Gastroenteritis and colitis of unspecified origin
A41.9	Sepsis, unspecified
B34.2	Coronavirus infection, unspecified site
B34.9	Viral infection, unspecified
B97.4	Respiratory syncytial virus as the cause of diseases classified to other chapters
H65.1	Other acute nonsuppurative otitis media
H66.0	Acute suppurative otitis media
H66.9	Otitis media, unspecified
J00.9	Acute nasopharyngitis
J01.2	Acute ethmoidal sinusitis
J01.4	Acute pansinusitis
J01.9	Acute sinusitis, unspecified
J02.9	Acute pharyngitis, unspecified
J03.0	Streptococcal tonsillitis
J03.9	Acute tonsillitis, unspecified
J04.0	Acute laryngitis
J04.1	Acute tracheitis
J04.2	Acute laryngotracheitis
J05.0	Acute obstructive laryngitis
J06.9	Acute upper respiratory infection, unspecified
J09.9	Influenza due to identified zoonotic or pandemic influenza virus
J10.0	Influenza with pneumonia, seasonal influenza virus identified
J10.1	Influenza with other respiratory manifestations, seasonal virus identified
J10.8	Influenza with other manifestations, seasonal influenza virus identified
J11.0	Influenza with pneumonia, virus not identified
J11.1	Influenza with other respiratory manifestations, virus not identified
J11.8	Influenza with other manifestations, virus not identified
J12.1	Respiratory syncytial virus pneumonia
J12.8	Other viral pneumonia
J12.9	Viral pneumonia, unspecified
J13.9	Pneumonia due to S. pneumoniae
J15.9	Bacterial pneumonia, unspecified
J18.0	Bronchopneumonia, unspecified
J18.1	Lobar pneumonia, unspecified
J18.9	Pneumonia, unspecified

© 2023 Hedberg P et al. *JAMA Pediatrics*.

J20.1	Acute bronchitis due to Haemophilus influenzae
J20.5	Acute bronchitis due to respiratory syncytial virus
J20.6	Acute bronchitis due to rhinovirus
J20.9	Acute bronchitis, unspecified
J21.0	Acute bronchitis due to respiratory syncytial virus
J21.8	Acute bronchiolitis due to other specified organisms
J21.9	Acute bronchitis, unspecified
J22.9	Unspecified acute lower respiratory infection
J39.8	Other specified diseases of upper respiratory tract
J45.1A	Asthma bronchiale, nonallergic asthma, Acute, infectious
J96.0	Acute respiratory failure
J96.9	Respiratory failure, unspecified
J98.7	Respiratory infections, not elsewhere classified
R05.9	Cough
R06.0	Dyspnoea
R06.2	Wheezing
R06.8	Other and unspecified abnormalities of breathing
R07.4	Chest pain, unspecified
R09.8	Other specified symptoms and signs involving the circulatory and respiratory syndrome
R11.9	Nausea and vomiting
R11.9B	Vomiting
R23.0	Cyanosis
R46.4	Slowness and poor responsiveness
R50.8	Other specified fever
R50.9	Fever, unspecified
R53.9	Malaise and fatigue
R65.1	Systemic Inflammatory Response Syndrome of infectious origin with organ failure
U07.1	COVID-19, virus identified
U07.2	COVID-19, virus not identified
U08.9	Personal history of COVID-19, unspecified
U10.9	Multisystem inflammatory syndrome associated with COVID-19, unspecified

Abbreviations: ICD-10=International Statistical Classification of Diseases and Related Health Problems 10th Revision

eTable 2. Inpatient ICD-10 codes used for classification of hospital admission

ICD-10 diagnosis code	Diagnosis name
A08.4	Viral intestinal infection, unspecified
A09.0	Other and unspecified gastroenteritis and colitis of infectious origin
A09.9	Gastroenteritis and colitis of unspecified origin
A41.0	Sepsis due to Staphylococcus aureus
A41.9	Sepsis, unspecified
B34.9	Viral infection, unspecified
H66.0	Acute suppurative otitis media
H66.9	Otitis media, unspecified
I40.9	Acute myocarditis, unspecified
J00.9	Acute nasopharyngitis
J01.2	Acute ethmoidal sinusitis
J01.4	Acute pansinusitis
J01.9	Acute sinusitis, unspecified
J03.0	Streptococcal tonsillitis
J03.9	Acute tonsillitis, unspecified
J04.0	Acute laryngitis
J04.1	Acute tracheitis
J04.2	Acute laryngotracheitis
J05.0	Acute obstructive laryngitis
J06.9	Acute upper respiratory infection, unspecified
J10.0	Influenza with pneumonia, seasonal influenza virus identified
J10.1	Influenza with other respiratory manifestations, seasonal virus identified
J10.8	Influenza with other manifestations, seasonal influenza virus identified
J11.8	Influenza with other manifestations, virus not identified
J12.1	Respiratory syncytial virus pneumonia
J12.8	Other viral pneumonia
J12.9	Viral pneumonia, unspecified
J13.9	Pneumonia due to S. pneumoniae
J15.9	Bacterial pneumonia, unspecified
J18.0	Bronchopneumonia, unspecified
J18.1	Lobar pneumonia, unspecified
J18.9	Pneumonia, unspecified
J20.5	Acute bronchitis due to respiratory syncytial virus
J20.6	Acute bronchitis due to rhinovirus
J20.9	Acute bronchitis, unspecified
J21.0	Acute bronchitis due to respiratory syncytial virus
J21.1	Acute bronchitis due to human metapneumovirus

© 2023 Hedberg P et al. *JAMA Pediatrics*.

J21.9	Acute bronchitis, unspecified
J45.1A	Asthma bronchiale, nonallergic asthma, Acute, infectious
J96.0	Acute respiratory failure
J96.09	Acute respiratory failure, unspecified type
M30.3	Mucocutaneous lymp node syndrome (Kawasaki)
R05.9	Cough
R06.0	Dyspnoea
R06.8	Other and unspecified abnormalities of breathing
R50.8	Other specified fever
R50.9	Fever, unspecified
R56.0	Febrile convulsions
U07.1	COVID-19, virus identified
U10.9	Multisystem inflammatory syndrome associated with COVID-19, unspecified

Abbreviations: ICD-10=International Statistical Classification of Diseases and Related Health Problems 10th Revision

© 2023 Hedberg P et al. *JAMA Pediatrics*.

eTable 3. Study definitions

Variable	Missing data	Definition	Time period	Possible values
**Study outcomes**				
Hospital admission	No	Admission to the hospital any time from day of ED visit and 14 days onwards. Only hospital admissions with an ICD-10 main diagnosis code indicative of a respiratory virus infection were considered (see eTable 2).	0 to 14 days from day of ED visit	Yes, No
ICU admission	No	Admission to the ICU during a hospital admission as defined above.	Entire hospitalization	Yes, No
30-day all-cause mortality	No	Date of death (of any cause) any time from day of ED visit and 30 days onwards	0 to 30 days from day of ED visit	Yes, No
**Other collected variables**				
Sex	No	Sex of individual	Birth	Male, Female
Age	No	Age at ED visit	Date of ED visit	0 to 17 years
Asthma	No	**ICD-10:** J45.X, J46.X	Fourteen days to five years before ED visit	Yes, No
Cancer	No	**ICD-10:** All codes from C00.X to C97.X (besides C44.X), Z51.0, Z51.1<br><br>**KVÅ:** DT107, DT108, DT112, DT116, DT135, DV070, DV071, DV134	Fourteen days to one year before ED visit<br><br>Fourteen days to one year before ED visit	Yes, No
Cardiac disease	No	**ICD-10:** I05.X, I06.X, I07.X, I08.X, I20.X, I21.X, I22.X, I24.X, I25.X, I26.X, I27.X,	Fourteen days to five years before ED visit	Yes, No

© 2023 Hedberg P et al. *JAMA Pediatrics*.

		I28.X, I34.X, I35.X, I36.X, I37.X, I42.X, I44.X, I45.X, I46.X, I47.X, I48.X, I49.X, I50.X		
Chronic kidney disease	No	**ICD-10:** N03.X, N05.X, N07.X, N18.X, N19.X, N25.X, N26.X, N27.X<br><br>**ICD-10:** Z49.1, Z49.2 (should be registered at least twelve times during the time period)<br><br>**ICD-10:** Z99.2<br><br>**KVÅ:** DR016, DR024 (should be registered at least twelve times during the time period)	Fourteen days to five years before ED visit<br><br>Fourteen days to one year before ED visit<br><br>Fourteen days to one year before ED visit<br><br>Fourteen days to one year before ED visit	Yes, No
Chronic respiratory disease (not asthma)	No	**ICD-10:** J43.X, J44.X, J47.X, J60.X to J69.X, J70.X to J80.X, J81.X, J82.X, J83.X, J84.X, J92.X, J93.X, J94.X, J95.X, J96.X, J98.X, J99.X	Fourteen days to five years before ED visit	Yes, No
Congenital malformations and abnormalities	No	**ICD-10:** All codes from Q00.X to Q99.X	Fourteen days or more before ED visit	Yes, No
Diabetes (type 1 or 2)	No	**ICD-10:** E10.X, E11.X	Fourteen days to five years before ED visit	Yes, No
Immunocompromised state	No	**ATC:** H02AB.X (should be registered at least twice during the time period)<br><br>**ATC:** L01.X	Fourteen days to half a year before ED visit<br><br>Fourteen days to one year	Yes, No

© 2023 Hedberg P et al. *JAMA Pediatrics*.

	**ATC:** L04.X<br><br>**ICD-10:** B20.X, B21.X, B22.X, B23.X, B24.X, D57.0, D57.1, D80.X, D81.X, D82.X, D83.X, D84.X, Z94.0, Z94.1, Z94.2, Z94.3, Z94.4, Z94.8<br><br>**KVÅ:** DR04.1, DR04.2, DR04.4, DR04.6, DR04.7<br><br>**KVÅ:** H02AB.X (should be registered at least twice during the time period)<br><br>**KVÅ:** L01.X<br><br>**KVÅ:** L04.X	before ED visit<br><br>Fourteen days to half a year before ED visit<br><br>Fourteen days or more before ED visit<br><br>Fourteen days to three years before ED visit<br><br>Fourteen days to half a year before ED visit<br>Fourteen days to one year before ED visit<br>Fourteen days to half a year before ED visit	
Perinatal conditions	**ICD-10:** All codes from P00.X to P96.X	Fourteen days or more before ED visit	Yes, No
Hospital length of stay	Number of days from hospital admission to hospital discharge	Hospital admission to hospital discharge	0 to 35 days
High-flow nasal cannula	**KVÅ:** DG028	During ED visit, hospitalization or stay in ICU	Yes, No
BiPAP, CPAP, or NIV	**KVÅ:** DG001, DG007, DG023, DG027	During ED visit, hospitalization or stay in ICU	Yes, No
Mechanical ventilation	**KVÅ:** DG021	During ED visit, hospitalization or stay in ICU	Yes, No

© 2023 Hedberg P et al. *JAMA Pediatrics*.

Abbreviations: ATC=Anatomical Therapeutic Chemical, BiPAP=Bilevel positive airway pressure, CPAP=continuous positive airway pressure, ED=Emergency department, ICD-10= International Statistical Classification of Diseases and Related Health Problems 10th Revision; ICU=Intensive care unit, KVÅ=Klassifikation av vårdåtgärder (Swedish for classification of healthcare procedures), NIV=Non-invasive ventilation

© 2023 Hedberg P et al. *JAMA Pediatrics*.
