# Supplementary material for: Outcomes of Pediatric SARS-CoV-2 Omicron Infection vs Influenza and Respiratory Syncytial Virus Infections
Source: JAMA Pediatr. 2023 Dec 26;178(2):197–9. doi: 10.1001/jamapediatrics.2023.5734 (PMC10751651; doi:10.1001/jamapediatrics.2023.5734)
Supplement: Supplement 2. — Data Sharing Statement [file jamapediatr-e235734-s002.pdf]

# Data Sharing Statement

Hedberg. Outcomes of Pediatric SARS-CoV-2 Omicron Infection vs Influenza and Respiratory Syncytial Virus Infections. *JAMA Pediatr*. Published December 26, 2023.
doi:10.1001/jamapediatrics.2023.5734

**Data**
**Data available:** No

**Additional Information**
**Explanation for why data not available:** The individual participant data underlying this article were subject to ethical approval and cannot be shared publicly. Data from the deidentified administrative health registry are not freely available due to protection of the personal integrity of the participants.
